# Complete genomic sequencing of canine distemper virus with nanopore technology during an epizootic event

**DOI:** 10.1038/s41598-022-08183-3

**Published:** 2022-03-08

**Authors:** Zsófia Lanszki, Gábor E. Tóth, Éva Schütz, Safia Zeghbib, Miklós Rusvai, Ferenc Jakab, Gábor Kemenesi

**Affiliations:** 1 National Laboratory of Virology, Szentágothai Research Centre, University of Pécs, Pécs, 7624 Hungary; 2 Institute of Biology, Faculty of Sciences, University of Pécs, Pécs, 7624 Hungary; 3 Exo-Pet Állatgyógyászati Centrum, Budapest, 1078 Hungary; 4 Vet-Diagnostics Kft., Szolnok, 5000 Hungary

**Keywords:** Infectious-disease diagnostics, Virology

## Abstract

Canine distemper virus (CDV) endangers a wide range of wild animal populations, can cross species barriers and therefore representing a significant conservational and animal health risk around the globe. During spring to autumn 2021, according to our current estimates a minimum of 50 red foxes (*Vulpes vulpes*) died of CDV in Hungary, with CDV lesions. Oral, nasal and rectal swab samples were RT-PCR screened for Canine Distemper Virus from red fox carcasses. To investigate in more detail the origins of these CDV strains, 19 complete genomes were sequenced with a pan-genotype CDV-specific amplicon-based sequencing method developed by our laboratory and optimized for the Oxford Nanopore Technologies platform. Phylogenetic analysis of the complete genomic sequences and separately the hemagglutinin gene sequences revealed the role of the Europe lineage of CDV as a causative agent for the current epizootic. Here we highlight the growing importance of fast developing rapid sequencing technologies to aid rapid response activities during epidemics or epizootic events. We also emphasize the urgent need for improved surveillance of CDV, considering the epizootic capability of enzootic strains as reported in the current study. For such future efforts, we provide a novel NGS protocol to facilitate future genomic surveillance studies.

## Introduction

Canine distemper virus (CDV) is a significant viral pathogen affecting domestic and wild animal species worldwide^1^. CDV is highly prone to cross-species transmission between domestic and wildlife reservoir hosts, representing a significant OneHealth challenge on the wildlife-domestic animals interface^2,3^. CDV is an RNA virus which belongs to the *Paramyxoviridae* family in the *Morbillivirus* genus^4–6^. The viral genome is ~ 15 kb and encodes six structural proteins^7^. The virus is primarily transmitted among animals via various body fluids, such as respiratory droplets, ocular discharge, nasal discharge, saliva, urine and feces, including transmission with direct contact^8^. The hemagglutinin (H) gene is an attachment protein, it has a key role as a receptor-binding protein. Amino acid variations in the hemagglutinin protein that bind cellular SLAM (signaling lymphocyte activation molecule) are thought to be important in species specificity^7^. Several distinct genotypes are known and classified according to different hosts and geographical areas based on nucleotide sequence analysis of the hemagglutinin gene^6,7,9^. The Arctic-like, Europe and European Wildlife lineages were reported in Hungary previously^10–12^.

CDV is able to trigger epizootic events, with significant negative impact on wild animal populations around the world. Over the last 2 decades, Europe lineage of the CDV has caused a number of local epizootics among wild carnivores in Europe mostly among Red foxes (*Vulpes vulpes*) and European badgers (*Meles meles*)^13–19^. Highlighting its conservational relevance across a number of animal taxa, a large number of Baikal seals (*Pusa sibirica*) were infected with CDV in Lake Baikal between 1987 and 1988, most likely as a result from a spillover event from dogs^20,21^. Caspian seals (*Pusa caspica*) were also seriously affected by the Caspian lineage of CDV in epizootics occurring in the Caspian sea between 1997 and 2000^18,22^. In Africa, the CDV causes a serious problem among Lions (*Panthera leo*)^23–25^. CDV infected Black-footed ferrets (*Mustela nigripes*) in Wyoming state of the USA were also reported^26,27^. All these examples highlight the importance of this virus for nature conservation aspects in multiple continents and distant geographic areas.

Next generation sequencing (NGS) technologies are increasingly used by microbiological laboratories to detect and characterize pathogens^28^. NGS performance can be optimized for rapid sample preparation, real-time sequence analysis and complete genome sequencing of pathogens^29–33^. MinION (Oxford Nanopore Technologies, Oxford, UK), a leading NGS technology, allows rapid and efficient sequencing, as it was already demonstrated in the case of CDV^34^. Amlicon-based NGS sequencing of specific pathogens is a method for the fast detection and genomic characterization of targeted pathogens, allowing high-coverage rapid sequencing of genomic sequence information^35–38^.

Here we present a novel NGS-based technique for the rapid genomic characterization of CDV strains and demonstrate the feasibility of this method during the genomic epidemiological investigation of a most likely widespread epizootic event in Hungary among foxes. In 2021, approximately fifty foxes were diagnosed with Canine Distemper Virus in multiple regions of Hungary. Two dozen of these foxes were analyzed in our laboratory. Our aim was to investigate these cases using rapid sequencing application and understand the genomic characteristics of the epizootic CDV strains. We highlight the need for extensive surveillance of enzootic CDV strains to follow-up the genetic evolution and give a better picture of the genomic background mechanisms of recurring outbreaks in Europe.

## Results

### PCR detection and sequencing

Carcasses of 6/5 cubs and 1/1 adult fox from the spring period, and 5/3 cubs 10/10 juvenile and 2/2 adult foxes from the summer period were positive for CDV with RT-PCR. A total of 21 of the 24 foxes tested were positive. After the results, 19 samples were selected for further sequencing, based on their low Ct (correlates with higher viral load) during the real-time RT-PCR reaction (Table 1). Finally, 19 complete CDV genomes were sequenced from foxes with a pan-genotype CDV-specific amplicon-based sequencing method resulting in high sequencing coverage (Supplementary Information 1). We retrieved the complete genomic data of all 19 samples and submitted these to GenBank (NCBI) database.

**Table 1:** Sequencing and diagnostic parameters of the investigated fox samples.

Accession number	Season	Age Category	RT-qPCR Ct value	Number of multiplex PCR cycles	Processed and mapped reads	Mean coverage on the targeted region (reads)
OK557779	Summer	Cub	21.50	25	19,846	1868.1
OK557780	Summer	Cub	24.15	25	14,192	1347.4
OK557781	Summer	Juvenile	29.25	28	14,244	1398.3
OK557782	Summer	Cub	26.02	28	14,491	1231.2
OK557783	Summer	Juvenile	27.12	28	12,588	1090.2
OK557784	Summer	Juvenile	25.46	28	6513	579.0
OK557785	Summer	Adult	36.90	36	14,186	1263.0
OK557786	Summer	Juvenile	27.83	28	11,827	1011.4
OK557787	Summer	Juvenile	38.58	36	10,750	1102.3
OK557788	Summer	Juvenile	28.15	28	8871	810.3
OK557789	Summer	Adult	38.25	36	4485	320.2
OK557790	Summer	Juvenile	28.32	28	10,203	1114.6
OK557791	Summer	Juvenile	26.74	28	8494	801.7
OK557792	Summer	Juvenile	22.83	25	7461	674.2
OK557793	Summer	Juvenile	21.49	25	12,266	1232.6
OK557794	Spring	Cub	31.33	32	93,228	5662.0
OK557795	Spring	Cub	31.91	33	63,959	3984.3
OK557796	Spring	Cub	30.78	32	56,054	3460.9
OK557797	Spring	Adult	32.69	33	80,721	4696.6

Most relevant next-generation sequencing quality data as the mapped reads and mean coverage per sample is presented. Number of multiplex PCR cycles is relevant for the amplicon-based NGS sequencing workflow.

The dog samples collected at the rescue center were tested negative for CDV.

### Phylogenetic analysis and amino acid differences in the H gene proteins

**Figure 1:**
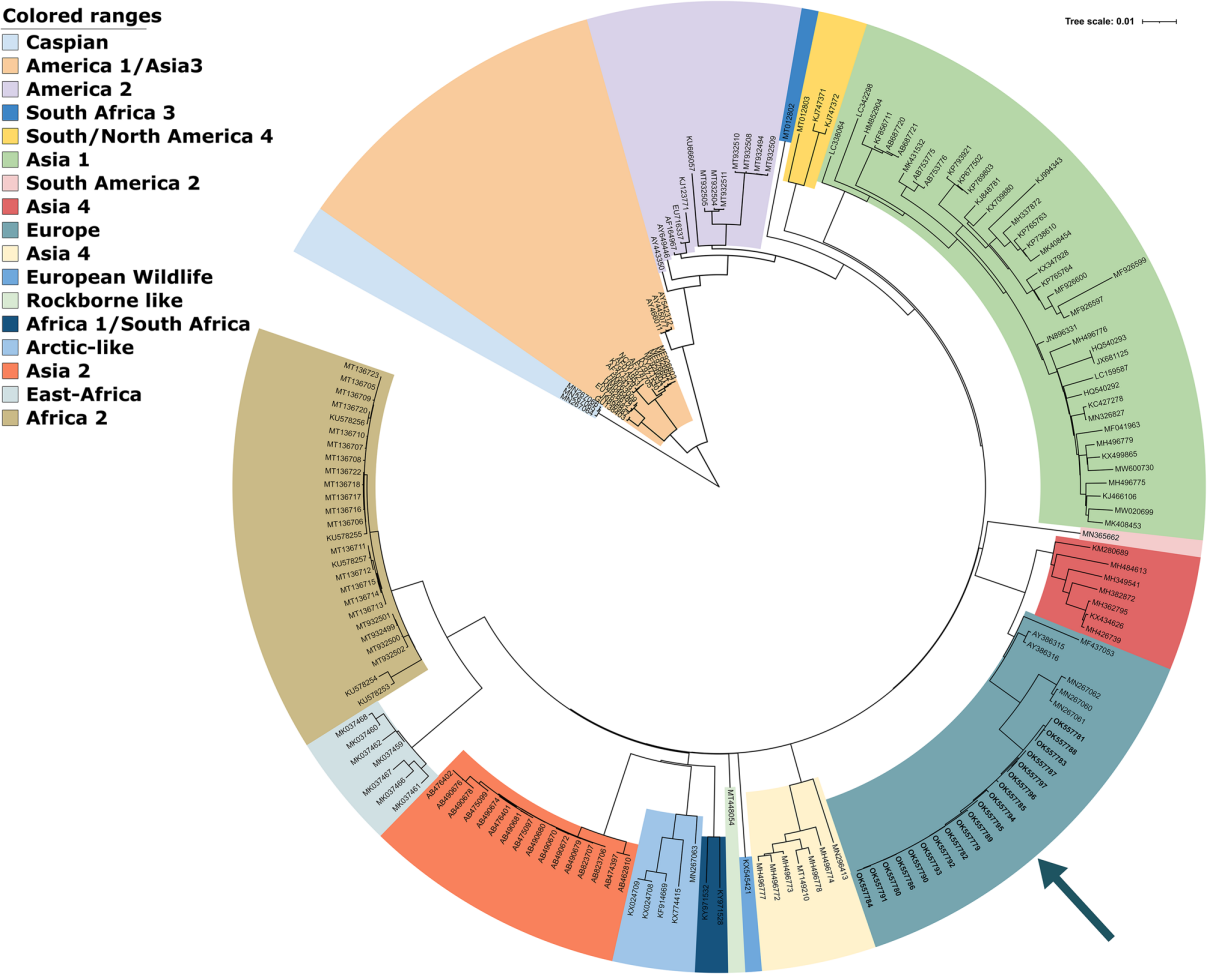
Maximum Likelihood phylogenetic tree based on 179 CDV complete genomes nucleotide sequences. Phocine distemper virus (PDV) (GenBank accession number: KY629928) was used as an outgroup to root the phylogenetic tree. The Europe lineage of interest is highlighted in light blue.

**Figure 2:**
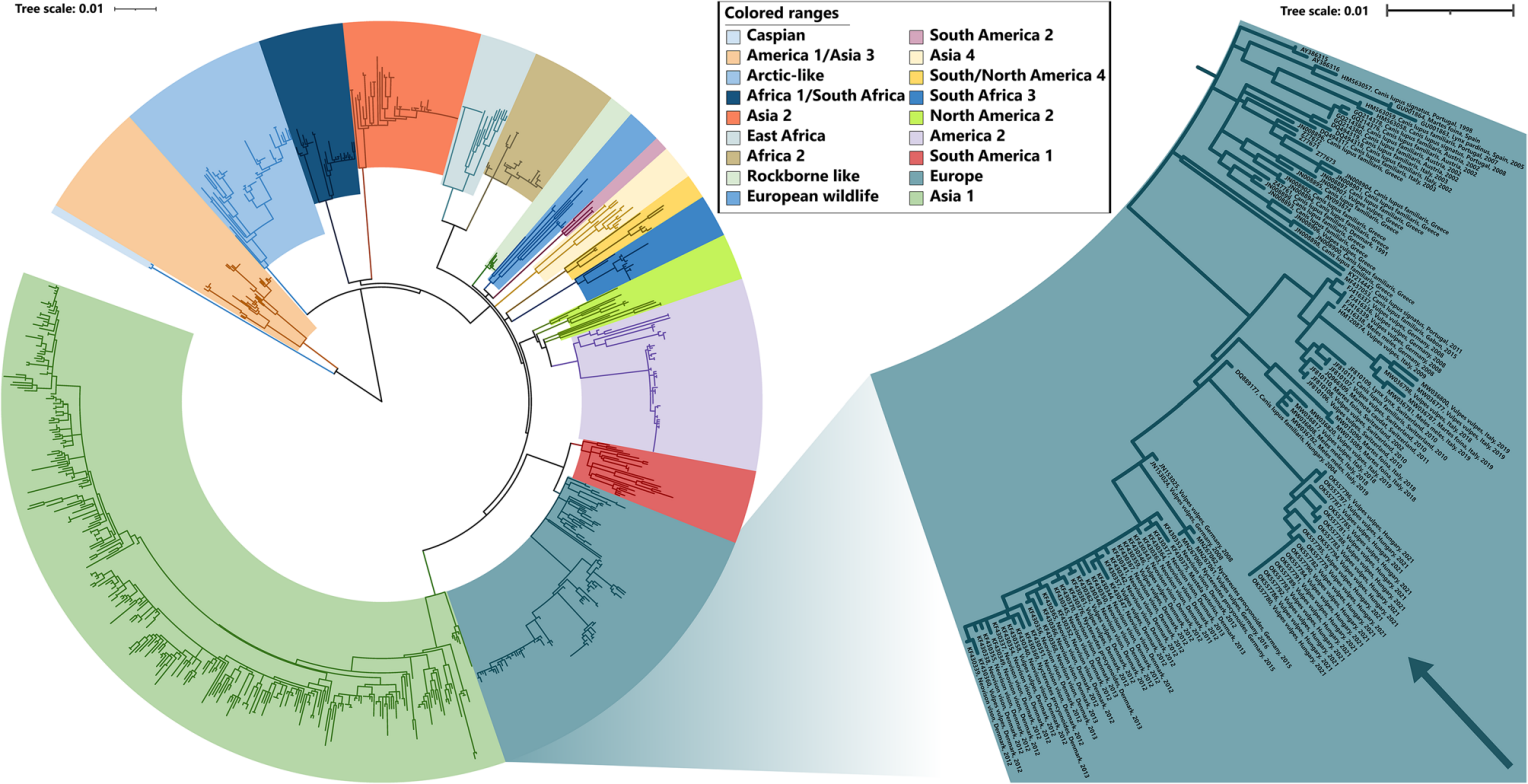
Maximum Likelihood phylogenetic tree based on 846 complete hemagglutinin (H) nucleotide sequences. Phocine distemper virus (PDV) (GenBank accession number: KY629928) was used as an outgroup to root the phylogenetic tree. The Europe lineage of interest is highlighted in light blue.

Based on the phylogenetic analysis of all currently recognized genotypes (Figs. 1 and 2), the CDV strains of this study belong to the Europe lineage. They are positioned in the genetic cluster of previously reported CDV sequences in Europe. We present the closest relation of the epizootic sequence cluster to a Hungarian dog sample from 2004, that is a unique branch within the Europe lineage of CDV and sequences of foxes from Germany in 2008, however the node connecting to this sequence cluster clearly indicates the lack of sequence data from previous years (Fig. 2). Therefore the source of the current epizootic strain remains unknown, nevertheless we present the closest genetic relation to regional sequences, supporting the epizootic potential of locally circulating CDV strains. Phylogenetic analysis of both the complete genome and the H protein sequence clearly showed that the current epizootic sequences are genetically related to the enzootic Europe genetic lineage of CDV.

All of the 19 H gene sequences from foxes contained G at position 530 and Y at position 549 which correlates with the constellation in the CDV sequence from a dog in Hungary, 2004, the closest known relative to the current epizootic strains.

## Discussion

We present the genomic sequencing and phylogenetic analysis of 19 complete genomes of CDV strains from red foxes during an epizootic event in Hungary, in 2021. We provided novel, complete genomic sequence data and showed the reliability of NGS sequencing in genomic epidemiological studies which may support rapid response actions during future epizootic situations. A total of 21 of the 24 foxes were positive for CDV with one-step RT-PCR. As reported by the animal rescue center, the 3 negative animals died with similar symptoms as the other 21, so it is conceivable that it was not possible to detect viral RNA in the collected swab samples due to viral clearance ^39–41^. In addition to the observed symptomatic animals, there may have been more undetected cases in the region. Notably, a limitation of our study is the lack of source information about the investigated animals. However, the phylogenetic relatedness and the elevated case number as experienced by the rescue center supports the idea of a more widespread epizootic event. Based on the phylogenetic analysis the sequences from the foxes belonged to the Europe lineage and showed the greatest similarity with an H gene of CDV which was detected in Hungarian dog sample from 2004 in the same area^10^.

Across Europe, episodes of canine distemper outbreaks in non-dog host species with Europe lineage have been reported earlier. In Germany, numerous wild red foxes exhibited neurological signs suggestive of canine distemper and several badgers were found dead. After H gene of CDV sequences were analyzed from five foxes and one badger were confirmed with Europe lineage from 2008^14^. In Italy, similar to the current epidemic in Hungary, at least 30 foxes with altered behavior were seen near human habitations and facilities in 2009. Most foxes were juveniles during the epizootic. Then the presence of the European lineage in three infected foxes was confirmed by H gene sequencing^13^. In Switzerland, numerous wild carnivores, including red foxes, Eurasian badgers, stone and pine martens, and one Eurasian lynx were found with CDV lesions between 2009 and 2010. The first 50 animals were confirmed as CDV positive. This outbreak was detected in a large spectrum of affected species, with high morbidity and mortality, especially among red foxes and badgers^15^. In Denmark, a major outbreak of canine distemper virus was detected in farmed minks (*Neovison vison*) from mink farms and a high number of species such as foxes, raccoon dogs (*Nyctereutes procyonoides*), and wild ferret (*Mustela putorius*), between 2012 and 2013^16^. The Europe lineage of CDV in wildlife has continued to be reported from nearby countries, first in Italy from wild animals, mainly foxes and badgers, between 2006 and 2009^17^, thereafter in Germany from raccoons (*Procyon lotor*) from 2015 and fox from 2016^18^, and recently in Northern Italy from foxes, badgers, and stone martens between 2018 and 2019^19^. Based on these epizootic events, it can be assumed that this lineage will be present among European wild animals, with recurring outbreaks in the future.

Understanding the evolution of enzootic strains and the transmission risk from wildlife to domestic animals are highly important to mitigate the effect of spillover events on household animals. During the last years, several studies recognized the importance of providing genetic data^42–45^. Host jump events from wildlife to domestic animals were supposedly connected to substitutions at the amino acid positions 530 and 549 in the signaling lymphocytic activation molecule SLAM binding region. It was hypothesized in multiple studies that the substitutions at the residues G/E 530 to R/D/N and Y549H may have a crucial role in the inter-species transmission from domestic dogs to non-dog hosts^2^. In contrast, the current study reports that all epizootic sequences from red foxes presented the 530G and 549Y, at the amino acid level. In this term, other studies support our current observations, since the CDV hemagglutinin gene sequences of red foxes in Germany, Denmark and Italy contain a 549H and a 549Y amino acid, indicating that both versions were found in red foxes^2,13,14,16^. Based on the data available so far, it needs to be reconsidered whether these amino acid substitutions and constellations correspond to the host or not.

The importance of sequencing data to better understand CDV evolution is increasingly recognized in other studies as well. Apart from the limitation of our study, namely the lack of different CDV lineages to extensively verify the method, we present a novel NGS-based sequencing performance to aid future studies. We designed the method to be applicable for sequencing multiple genetic lineages. Next-generation sequencing methods were previously used in relation to CDV research. In a study, CDV infection was identified in a dog that was imported to Italy from Cuba. CDV was detected and isolated from the infected brain tissue. Subsequently, this isolate was subjected for non-targeted Next-Generation Sequencing using the MinION Nanopore technology^34^. Another recent study presented complete genomic data which was acquired by Sanger sequencing method. These papers well represent the increasing need for rapid and specific genomic data generation^46^. Using the amplicon-based NGS sequencing technology is not unique in epidemic situations, but it was fairly used in veterinary health-related events to date. We highlight the importance of similar methods to aid future investigations of epizootic events or even supporting surveillance efforts. In addition, as presented on the phylogenetic analysis, there is a significant lack of genetic sequence information about enzootic and non-enzootic CDV strains. However we designed the application to be specific for several genetic lineages, we were able to sequence only Europe lineage CDV samples within the framework of this study. The NGS workflow of the current study needs to be tested on other genetic lineages of CDV as well in the future.

From a nature conservation point of view, it is of paramount importance to learn more about diseases in animal species susceptible to CDV infection and prepare or aid mitigation efforts during epizootic events. Foxes’ social behavior during the reproductive season and the dispersion of juvenile animals can played a major role in epizootic CDV amplification and diffusion in a wide geographic range, as discussed before^13,19^. CDV is known to easily cross species barriers and is able to infect different animal species. Notably, to better understand recurring epizootics of enzootic CDV strains needs the perspective of OneHealth concept. We need to better understand environmental and animal behavioral factors, among many others.

## Methods

### Case history and sample collection from red foxes

Animal samples were collected opportunistically as part of the veterinary investigation of symptomatic cases at the animal rescue center. After official veterinary diagnostic procedures the samples for this study were additionally collected by the veterinary practitioner. All methods were carried out in accordance with relevant guidelines and regulations. At the end of the winter of 2021, the first reports of wild living red foxes with CDV symptoms arrived. Between spring and late summer, animal rescuers registered a minimum of 50 cases across the country. Most of the animals were cubs or juveniles at this period. Of these 50 animals, carcasses of 6 cubs and 1 adult fox were obtained during the spring period, and an additional 5 cubs, 10 juveniles and 2 adult foxes during the summer period. Samples were obtained for laboratory examination after the fatal outcome of the disease. Fox carcasses were stored at − 20 °C at the veterinary clinic. During sampling, oral, nasal, and rectal swabs were collected with sterile sampling stick into one tube per animal.

Symptomatic live foxes were also sampled with oral and nasal swabs at the rescue center. Sampling was conducted by the veterinary practitioner. Although the foxes were quarantined from the dogs living in the rescue center, saliva samples were taken from the dogs (n = 5) as well. The dogs had no symptoms during the season.

All samples were received at the request of the animal rescue center, to investigate the origin of the CDV strain.

### PCR reaction

All swabs were homogenized in 500 µl of Phosphate-buffered saline (PBS). 100 µl of the supernatant was used for RNA extraction using the Monarch total RNA miniprep kit (NEB, USA). The samples were tested using a CDV-specific real-time RT-PCR as previously described, with some modifications^5^. All PCRs were performed using OneStep RT-PCR Kit (Qiagen, Germany) at 50 °C for 30 min, and 95 °C for 15 min, followed by 45 cycles of 95 °C for 20 s, 46 °C for 30 s 72 °C for 30 s (the fluorescence signal was detected during the annealing step). All PCRs were run on the MyGo Pro PCR system platform (IT-IS Life Science, Ireland). RT-PCRs were performed immediately after RNA extraction without freeze-thawing the nucleic acid, avoiding RNA degradation.

### MinION library preparation, sequencing and data analysis

The complete genome sequencing was performed with MinION nanopore sequencing technology (Oxford Nanopore Technologies, UK). We developed an amplicon-based sequencing method based on previous protocols ^47,48^. cDNA preparation from the CDV positive RNA sample was conducted with Superscript IV (Invitrogen, USA) using random hexamers. Genome-specific, overlapping amplicons were generated from cDNA with the Q5 Hot Start HF Polymerase (New England Biolabs, USA) with multiple primer sets in parallel pools (CDV_1000bp_pool_1:63 primers, CDV_1000bp_pool_2:50 primers, CDV_2000bp_pool_1:32 primers, CDV_2000bp_pool_2:27 primers (Supplementary Informations 2 and 3). Following the amplicon PCR DNA from the same primer set (1000 or 2000) were purified with AMPure XP beads (Beckman Coulter, USA) as per manufacturer’s instructions. The end-repair and dA tailing were performed with the NEBNext Ultra II End Repair/dA-Tailing Module (New England Biolabs, USA). End-prepped DNA were transferred to the next reaction directly and the barcode derived from EXP-NBD196 (Oxford Nanopore Technologies, UK) were ligated with NEBNext Ultra II Ligation Module (NEB, USA). After, the pooled barcoded samples were jointly cleaned up with Ampure XP beads, the AMII sequencing adapters were ligated with NEBNext Quick Ligation Module. The final library was quantified with Qubit dsDNA HS Assay Kit (Invitrogen, USA) on Qubit 3 fluorometer. 60 ng of the final library was loaded onto a R9.4.1 (FLO-MIN106D) flow cell. The detailed protocol is available at our laboratory protocols.io page^49^.

We ran the ONT guppy software under Ubuntu Linux 18.04. Base-calling with super-accuracy basecaller algorithm (dna_r9.4.1_450bps_sup config file), were carried out with Guppy basecaller (version 5.0.7.). Demultiplexing and trimming of barcodes were performed with Guppy using default parameters of “guppy_barcoder” runcode. The demultiplexed reads were length filtered when reads under 800 basepair in the case of 1000 primer set and under 1800 basepair by the 2000 primer set were eliminated from the dataset. Additional 50 basepair were trimmed from the both ends of the reads and were mapped to the MN267060 to generate preconsensus with the usage of Genious mapper (version Geneious Prime 2021.2.2). Medaka (version 1.4.2) was used to map trimmed reads against the preconsensus to generate polished consensus sequences. The generated consensus sequences were manually checked for base-calling errors especially in the homopolymeric regions.

### Phylogenetic analysis

Sequences for both datasets (complete genomes and H genes) were first aligned in MAFFT webserver using default parameters. Thereafter, IQTREE webserver was used for both best substitution model selection and maximum likelihood phylogenetic tree reconstruction using ultrafast bootstrapping. The complete genomes phylogenetic tree analysis was performed under the GTR + F + I + G4 substitution model chosen according to Bayesian Information Criterion (BIC). Whereas, the H gene phylogeny was implemented under the TVM + F + I + G4 according to BIC. Phocine distemper virus (PDV) was used as an outgroup to root both phylogenetic trees. Subsequently, trees were edited in iTOL webserver.

The H gene has one open reading frame encoding 607 amino acids, which amino acids differences between the H gene from foxes and at positions 530 and 549 were examined manually in the MAFFT alignment.

## Supplementary Information

Supplementary Information 1.

Supplementary Information 2.

Supplementary Information 3.
