# Supplementary material for: Complete genomic sequencing of canine distemper virus with nanopore technology during an epizootic event
Source: Sci Rep. 2022 Mar 8;12:4116. doi: 10.1038/s41598-022-08183-3 (PMC8904823; doi:10.1038/s41598-022-08183-3)
Supplement: Supplementary file 1 — Supplementary Information 1. [file 41598_2022_8183_MOESM1_ESM.pdf]

Supplementary Information

Complete genomic sequencing of Canine Distemper virus with Nanopore technology during an epizootic event

Zsófia Lanszki[1,2], Gábor E. Tóth[1,2], Éva Schütz[3], Safia Zeghbib[1,2], Miklós Rusvai[4], Ferenc Jakab[1,2], Gábor Kemenesi[1,2]

[1] National Laboratory of Virology, Szentágothai Research Centre, University of Pécs, 7624 Pécs, Hungary

[2] Institute of Biology, Faculty of Sciences, University of Pécs, 7624 Pécs, Hungary

[3] Exo-Pet Állatgyógyászati Centrum, 1078 Budapest, Hungary

[4] Vet-Diagnostics Kft., 5000 Szolnok, Hungary

* Correspondence: kemenesi.gabor@gmail.com

Supplementary Information: Figure 1. Visualization of sequencing coverage via the amplicon-based sequencing method for each sample. Horizontal scale represents the genomic position, whilst the vertical scale displays the coverage values of the sequencing reaction.

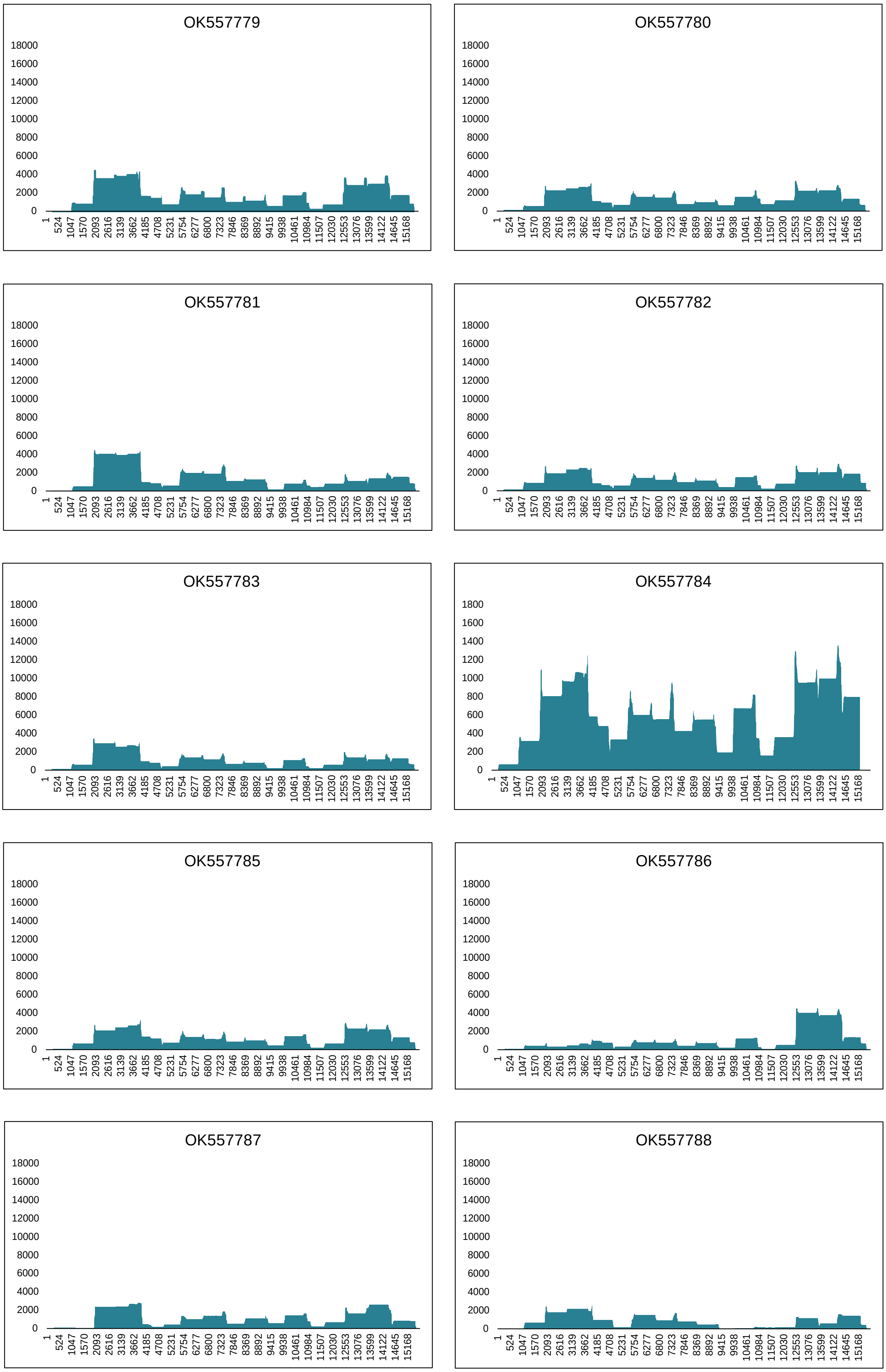
OK557779
18000 16000 14000 12000 10000 8000 6000 4000 2000 0
1 524 1047 1570 2093 2616 3139 3662 4185 4708 5231 5754 6277 6800 7323 7846 8369 8892 9415 9938 10461 10984 11507 12030 12553 13076 13599 14122 14645 15168
OK557780
18000 16000 14000 12000 10000 8000 6000 4000 2000 0
1 524 1047 1570 2093 2616 3139 3662 4185 4708 5231 5754 6277 6800 7323 7846 8369 8892 9415 9938 10461 10984 11507 12030 12553 13076 13599 14122 14645 15168
OK557781
18000 16000 14000 12000 10000 8000 6000 4000 2000 0
1 524 1047 1570 2093 2616 3139 3662 4185 4708 5231 5754 6277 6800 7323 7846 8369 8892 9415 9938 10461 10984 11507 12030 12553 13076 13599 14122 14645 15168
OK557782
18000 16000 14000 12000 10000 8000 6000 4000 2000 0
1 524 1047 1570 2093 2616 3139 3662 4185 4708 5231 5754 6277 6800 7323 7846 8369 8892 9415 9938 10461 10984 11507 12030 12553 13076 13599 14122 14645 15168
OK557783
18000 16000 14000 12000 10000 8000 6000 4000 2000 0
1 524 1047 1570 2093 2616 3139 3662 4185 4708 5231 5754 6277 6800 7323 7846 8369 8892 9415 9938 10461 10984 11507 12030 12553 13076 13599 14122 14645 15168
OK557784
1800 1600 1400 1200 1000 800 600 400 200 0
1 524 1047 1570 2093 2616 3139 3662 4185 4708 5231 5754 6277 6800 7323 7846 8369 8892 9415 9938 10461 10984 11507 12030 12553 13076 13599 14122 14645 15168
OK557785
18000 16000 14000 12000 10000 8000 6000 4000 2000 0
1 524 1047 1570 2093 2616 3139 3662 4185 4708 5231 5754 6277 6800 7323 7846 8369 8892 9415 9938 10461 10984 11507 12030 12553 13076 13599 14122 14645 15168
OK557786
18000 16000 14000 12000 10000 8000 6000 4000 2000 0
1 524 1047 1570 2093 2616 3139 3662 4185 4708 5231 5754 6277 6800 7323 7846 8369 8892 9415 9938 10461 10984 11507 12030 12553 13076 13599 14122 14645 15168
OK557787
18000 16000 14000 12000 10000 8000 6000 4000 2000 0
1 524 1047 1570 2093 2616 3139 3662 4185 4708 5231 5754 6277 6800 7323 7846 8369 8892 9415 9938 10461 10984 11507 12030 12553 13076 13599 14122 14645 15168
OK557788
18000 16000 14000 12000 10000 8000 6000 4000 2000 0
1 524 1047 1570 2093 2616 3139 3662 4185 4708 5231 5754 6277 6800 7323 7846 8369 8892 9415 9938 10461 10984 11507 12030 12553 13076 13599 14122 14645 15168

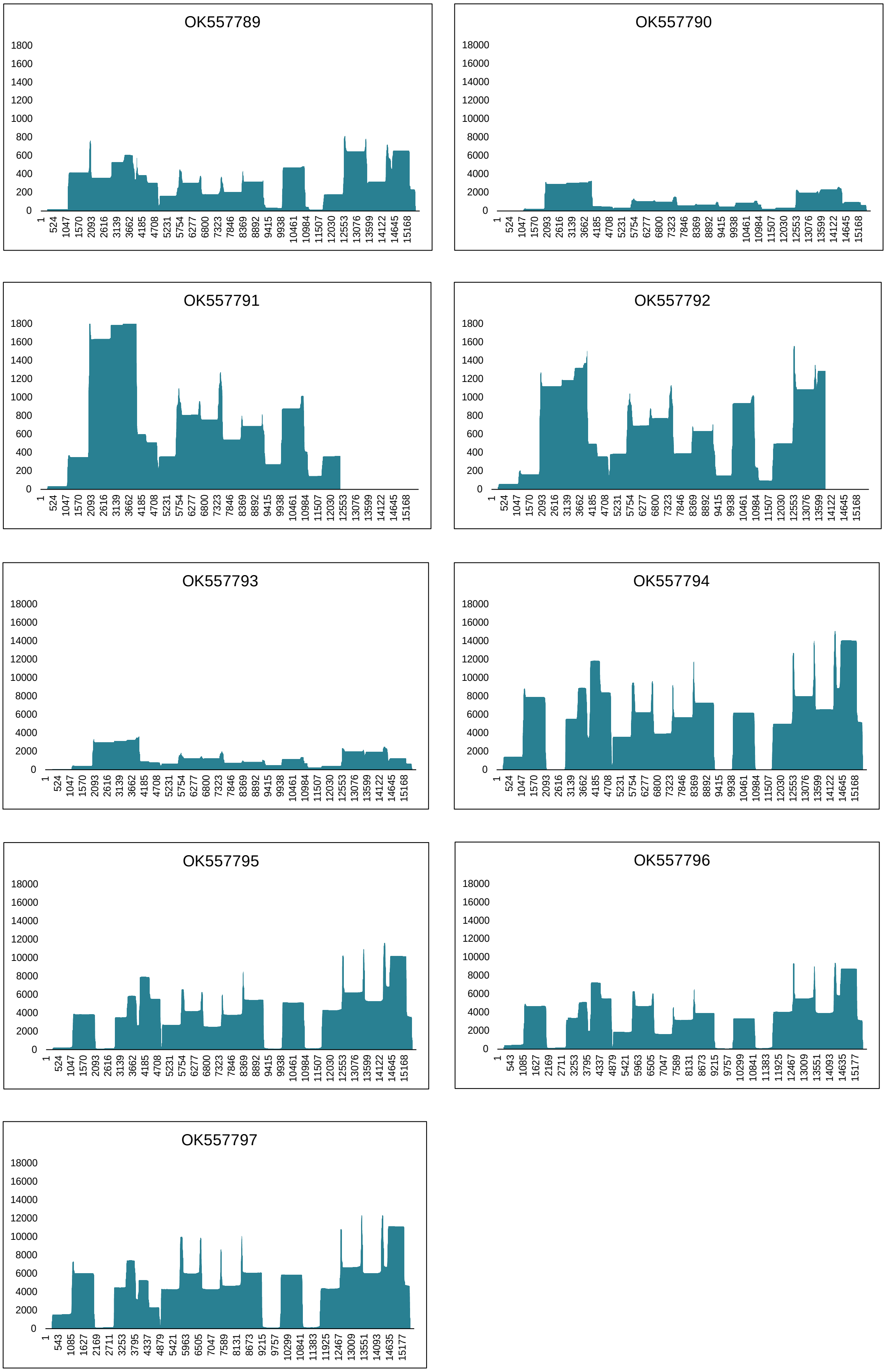
OK557789
1800 1600 1400 1200 1000 800 600 400 200 0
1 524 1047 1570 2093 2616 3139 3662 4185 4708 5231 5754 6277 6800 7323 7846 8369 8892 9415 9938 10461 10984 11507 12030 12553 13076 13599 14122 14645 15168
OK557790
18000 16000 14000 12000 10000 8000 6000 4000 2000 0
1 524 1047 1570 2093 2616 3139 3662 4185 4708 5231 5754 6277 6800 7323 7846 8369 8892 9415 9938 10461 10984 11507 12030 12553 13076 13599 14122 14645 15168
OK557791
1800 1600 1400 1200 1000 800 600 400 200 0
1 524 1047 1570 2093 2616 3139 3662 4185 4708 5231 5754 6277 6800 7323 7846 8369 8892 9415 9938 10461 10984 11507 12030 12553 13076 13599 14122 14645 15168
OK557792
1800 1600 1400 1200 1000 800 600 400 200 0
1 524 1047 1570 2093 2616 3139 3662 4185 4708 5231 5754 6277 6800 7323 7846 8369 8892 9415 9938 10461 10984 11507 12030 12553 13076 13599 14122 14645 15168
OK557793
18000 16000 14000 12000 10000 8000 6000 4000 2000 0
1 524 1047 1570 2093 2616 3139 3662 4185 4708 5231 5754 6277 6800 7323 7846 8369 8892 9415 9938 10461 10984 11507 12030 12553 13076 13599 14122 14645 15168
OK557794
18000 16000 14000 12000 10000 8000 6000 4000 2000 0
1 524 1047 1570 2093 2616 3139 3662 4185 4708 5231 5754 6277 6800 7323 7846 8369 8892 9415 9938 10461 10984 11507 12030 12553 13076 13599 14122 14645 15168
OK557795
18000 16000 14000 12000 10000 8000 6000 4000 2000 0
1 524 1047 1570 2093 2616 3139 3662 4185 4708 5231 5754 6277 6800 7323 7846 8369 8892 9415 9938 10461 10984 11507 12030 12553 13076 13599 14122 14645 15168
OK557796
18000 16000 14000 12000 10000 8000 6000 4000 2000 0
1 543 1085 1627 2169 2711 3253 3795 4337 4879 5421 5963 6505 7047 7589 8131 8673 9215 9757 10299 10841 11383 11925 12467 13009 13551 14093 14635 15177
OK557797
18000 16000 14000 12000 10000 8000 6000 4000 2000 0
1 543 1085 1627 2169 2711 3253 3795 4337 4879 5421 5963 6505 7047 7589 8131 8673 9215 9757 10299 10841 11383 11925 12467 13009 13551 14093 14635 15177
